# Effect of Different Ameliorants on the Infiltration and Decontamination Capacities of Soil

**DOI:** 10.3390/ma16072795

**Published:** 2023-03-31

**Authors:** Tianyi Sang, Aihong Kang, Yao Zhang, Bo Li, Huiwen Mao, Heyu Kong

**Affiliations:** 1 College of Civil Science and Engineering, Yangzhou University, Yangzhou 225100, China; 2 Research Center for Basalt Fiber Composite Construction Materials, Yangzhou 225127, China

**Keywords:** green space soil, soil ameliorants, soil infiltration, soil decontamination

## Abstract

The expansion of urban construction areas can reduce the infiltration rate of rainwater in permeable land, and a large amount of runoff rainwater cannot penetrate the soil. In extreme rainstorm weather, it is easy to cause serious urban waterlogging problems. To improve the infiltration and decontamination ability of green space soil, two types of inorganic ameliorants (i.e., sand and grain shell) and structural ameliorants (i.e., desulfurization gypsum and polyacrylamide) were utilized as amendments in the soil. The influence of the selected ameliorants on the infiltration and decontamination ability was analyzed through a soil infiltration test, soil pore distribution determination and a soil decontamination test. Three parameters including the soil infiltration rate, pore distribution characteristics and pollutant removal rate were proposed. The results showed that sand, grain shells and desulfurization gypsum (FGD gypsum) all enhanced the infiltration capacity of soil, while PAM decreased the infiltration capacity. Meanwhile, mixed sand and grain shell with the FGD gypsum and polyacrylamide can effectively improve the decontamination capacity of the soil. Comprehensive analysis showed that the better improvement combination is 10% sand + 20% grain hull + 0.5 g/kg FGD gypsum + 0.1 g/kg PAM.

## 1. Introduction

Urban flooding and stormwater runoff pollution have become increasingly prominent with the acceleration of urbanization and the dramatic increase in impervious surfaces. Stormwater runoff pollution [1,2] has become the third-largest source of pollution leading to urban water pollution. Depressional soakaway systems [3,4,5] and multifunctional storage facilities have been used as common means of runoff storage in Germany, Japan, and other developed countries. As an indispensable part of the urban ecosystem, the green space system is an important factor in maintaining and improving urban stormwater and water pollution. Soil consists of a fundamental component of green space systems, with their infiltration and decontamination capacities, which play a crucial role in the water cycle and ecological functions of such areas [6]. The soil’s texture, capacity, porosity, and organic matter content have a major impact on these capacities [7]. Unfortunately, the flooding and pollution of green spaces are becoming increasingly common in some of China’s major cities, especially during heavy rainfall events [8,9,10]. In such situations, a lack of soil infiltration and decontamination capacities emerges as a key factor.

Adding ameliorants into the soil is a more economical way to make green space soil suitable for rainwater storage, infiltration and decontamination without replacing the site soil. Songrui Ning et al. [11] investigated the effect of polyacrylamide (PAM) and sodium carboxymethyl cellulose (CMC) on the infiltration performance of coarse-textured soils in an indoor experiment. It was found that the CMC better inhibited soil sorption. Luna Ramos et al. [12] used a factorial design combining compost, sewage sludge and a control group with mulches to restore the porosity and permeability of soils affected by mining activities. The results showed that organic amendments modified soil infiltration and reduced water erosion, and the woodchip mulch was effective in capturing runoff and sediment. Cheung et al. [13] used alkaline fly ash to amend the soil to purify phosphate (${PO}_{4}^{3-}$) during sewage infiltration, and the results show that 5–15% precipitator fly ash and less than 30% lagoon fly ash could inhibit ${PO}_{4}^{3-}$ well. Chen et al. [14] used peat as an amendment material to improve the infiltration system of subsurface wastewater through soil column tests. It was found that by adding peat to the lower section of the infiltration system, the removal rate of Total Nitrogen (TN) and Nitrate Nitrogen (${NO}_{3}^{-}-N$) reached 94.1%, and the denitrification efficiency was significantly improved. Wang et al. [15] used straw with different treatments combined with inorganic amendments to study the effect on the water-holding capacity of the soil. The experimental results showed that the addition of long straw greatly hindered the soil infiltration capacity, and the ammonia-crushed straw improved the soil structure more significantly than the crushed straw. The modified soil has excellent soil water-holding capacity. Several researchers, including Khan, Hamid, and Bashir [16,17,18], have used different organic amendments to amend soil contaminated with cadmium (Cd). It was shown that organic amendments going through adsorption and complexation reactions could mitigate Cd contamination. However, the heavy metal concentration of the soil should be characterized before application. Hodson et al. [19] added bone meal to the soil to mitigate metal contamination by forming phosphate. The scanning electron microscopy analysis was performed to demonstrate the feasibility of bone meal amendment within the soil. Malandrino et al. [20] conducted a potting experiment on soils contaminated with copper, chromium and nickel by Vermiculite. The study showed that the addition of vermiculite substantially reduced the uptake of pollutants by plants, confirming the possibility of vermiculite amending metal-contaminated soils. Gray et al. [21] conducted field experiments with lime and red mud as amendments on highly contaminated soils. The results showed that the red mud and lime could be used to remediate highly contaminated acidic soils. Generally, soil pollution can be divided into organic pollution and inorganic pollution [22]. Among inorganic pollution, heavy metal pollution [23,24] accounts for a larger proportion than other pollutions, which are more harmful to human beings. Hence, the commonly used amendments (i.e., sand and grain shells) were selected to modify the soil to find a better heavy metal pollution absorber. At the same time, the investigation found that structural amendments can affect the structure and pore space of the soil. The research on the effect of amendments interweaving on soil properties is limited.

This study aims to assess the infiltration and decontamination capacities of modified soils with different types of ameliorants. To achieve this, we employed a self-designed infiltration test device to calculate the cumulative infiltration and infiltration rate and evaluated the infiltration capacity of different modified soils using the Kostiakov two-parameter model. In addition, the pore distribution of the modified soils was analyzed by mercury intrusion porosimetry (MIP). To quantitatively evaluate the level of pollution in collected rainwater, we used the event mean concentration (EMC) and conducted decontamination tests on different modified soils by simulating runoff rainwater. The study introduces a novel approach to evaluating the infiltration and decontamination capacities of modified soil and proposing soil modification techniques that are tailored to the specific conditions of diverse regions. This research provides valuable insights for the development of urban green spaces and offers practical recommendations for future projects.

## 2. Materials and Methods

### 2.1. Materials

#### 2.1.1. Soil

The soil used in this study was taken from Hanjiang District, Yangzhou City, Jiangsu Province. The BT-9300H laser particle size distribution instrument was used to test the soil particle size distribution [25]. According to the International Standard for the classification of soil texture, the soil was classified as “Sandy soil”, and its properties are shown in Table 1.

#### 2.1.2. Ameliorants

**Figure 1:**
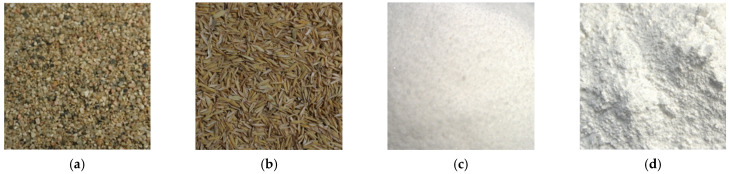
Macroscopic images of modified materials; (**a**) Sand; (**b**) Grain shells; (**c**) PAM; (**d**) FGD gypsum.

In this study, we utilized sand and grain shells as inorganic ameliorants. The grain shells were sourced from rice, while the sand was artificially treated from quartz sandstone. Both inorganic ameliorants were produced in Jiangsu Province, China. The FGD gypsum and polyacrylamide (PAM) were selected as structural ameliorants for improving the decontamination ability of the soil. The properties of the inorganic ameliorants and structural ameliorants are shown in Table 2, and the corresponding image is shown in Figure 1.

### 2.2. Experimental Protocol

The experimental design is shown in Table 3. First, the inorganic ameliorants (sand and grain shell) were added into the soil to study the influence of different inorganic ameliorants on the infiltration and decontamination capacity (A0, B1, B2, C1, C2). Therefore, this paper selected the inorganic ameliorant, which has better heavy metal removal ability, to further study the impact of the structural ameliorant (D1, D2, D3). Then, the control group (E1) was set up to study the effect of the interweaving of the inorganic ameliorants (sand and grain shell) and the structural ameliorant (PAM and FGD gypsum) on the water infiltration and decontamination ability of soil (E1, E2, E3, E4).

### 2.3. Test Methods

#### 2.3.1. Soil Infiltration Test

**Figure 2:**
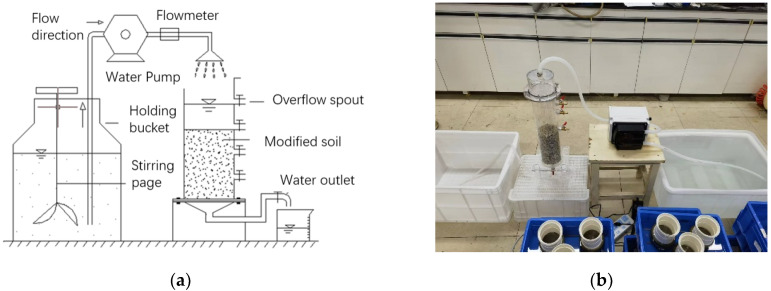
Diagram of the test setup: (**a**) Device construction diagram; (**b**) Actual installation diagram.

According to a study for determining soil infiltration capacity proposed by Bowles [26], the infiltration coefficients of the soils amended by different ameliorants were measured by a self-designed infiltration test device (Figure 2). Test parameters are controlled by a flow meter, which controls the water supply flow rate to simulate light rain patterns. The air-dried test soil was sieved through a 2 mm mesh to remove impurities and subsequently dried at 105 °C to its weight.

To ensure the consistent compaction of the soil samples, the soil was uniformly compacted to a height of 10 cm by applying two manual tampings. The bulk density of the original sample soil was measured to be 1.65 g/cm^3^, and the void ratio was 8.1%. Subsequently, the soil samples were submerged in water for a minimum of 24 h prior to testing, and the initial moisture content of the original soil was determined to be 7.9%. A head difference of 30 cm was established, and filter paper was affixed to both ends of the soil column to prevent soil loss. The rate of water discharge was measured at 1, 5, 10, 15, 20, 25, 30, 40, 50, 60, 80, 100, 120, and 150 min intervals following the initial outflow. The infiltration rate of the soil was determined using Equation (1) in this study.

(1) $$K=\frac{Q\cdot L}{A\cdot \Delta h\cdot t}$$

where *K* is the infiltration coefficient, cm^3^; *Q* is the water output, cm³; *L* is the height of the soil sample, cm; *A* is the cross-sectional area of the soil sample, cm²; Δ*h* is the head difference, cm.

As an important part of the conversion of rainwater, groundwater and soil water infiltration has important practical significance for the study of road runoff and soil water distribution. Based on Darcy’s law, many domestic and foreign scholars have proposed models such as Green–Ampt, Philip, Kostiakov and Richard for predicting the infiltration process of soil water. In this paper, the Kostiakov two-parameter infiltration model is used to analyze the infiltration capacity of soil with different ameliorants by using Equation (2).

$$I=Kt^{1-\alpha }$$

(2) lgI=lgK+(1−α)×lgt

lgI=P+β×lgt

where *I* is the cumulative infiltration, cm^3^; *t* is the infiltration time, min; *α* is the infiltration index; *P* is the initial infiltration index of soil; *β* is the infiltration decay rate parameters.

#### 2.3.2. Soil Pore Distribution Determination

Soil pores are essential for supporting plant growth and soil microbial activity. These pores are formed between soil particles, as well as within or between agglomerate structures [27,28]. Soil pores can store water and air while also providing a conduit for root growth and microbial movement. Measuring soil pore distribution is crucial for understanding soil–water relationships and predicting soil behavior.

**Figure 3:**
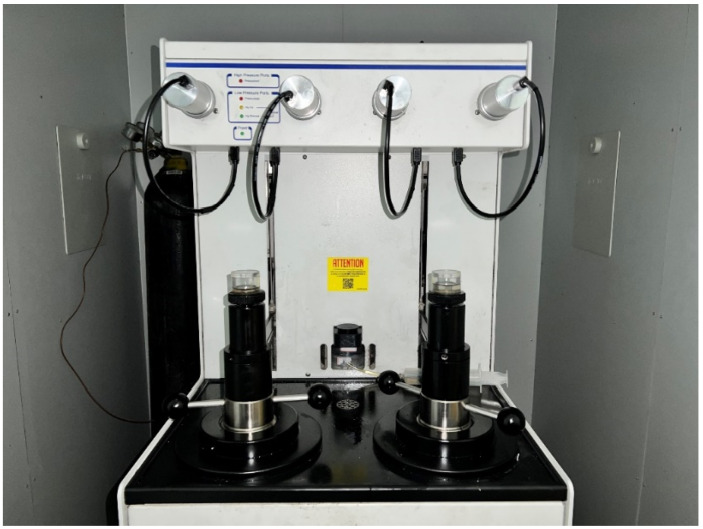
Mercury porosimeter.

Mercury intrusion porosimetry (MIP) is a powerful technique used to characterize soil pore structures. This method exploits the non-infiltration property of mercury (Hg) to measure pore size distribution under different applied pressures. The AutoPore 9500 mercury compactor (Micromeritics, Norcross, GA, USA) was employed in this study (Figure 3), with a maximum mercury pressure of 40,000 psia and a minimum void size of 5.5 µm.

To minimize the influence of sample preparation on pore distribution, the soil samples were standardized for tamping and cut into small pieces (5 mm in length). The samples were then vacuum freeze-dried before MIP analysis, following established operating procedures for the mercury compactor.

#### 2.3.3. Soil Decontamination Test

To evaluate the decontamination capacity of the different amended soils, this study sampled the typical runoff rainwater collected from Yangzhou City. The pollution level of the runoff rainwater was quantitatively evaluated using the Event Mean Concentration (EMC). To simulate the runoff rainwater, chemical reagents corresponding to different pollutant indicators were utilized, and the methods for detecting pollutant components, results and corresponding reagent choices are presented in Table 4.

Following the procedure for testing infiltration performance, the soils were dried, water-saturated and then standardized by tamping before being filled into the test device. A water flow rate of 2 mm/h was selected under the ASTM D2434-68 standard to simulate the light rain. After a two-minute treatment period, purified water samples were collected, and their pollutant content was measured. The obtained results were used to calculate the pollutant removal rate by applying Equation (3).

(3) $$\eta =\frac{C_{0}-C_{1}}{C_{0}}\times 100\%$$

where *COD* is the chemical oxygen demand; *TN* is the total Nitrogen; *TP* is the total Phosphorus; *Zn* is the zinc; *Pb* is the lead; *η* is the pollutant removal rate, %; *C*_0_ is the initial pollutant concentration, %; *C*_1_ is the pollutant concentration after purification, %.

## 3. Results and Discussion

### 3.1. Infiltration Capacity

**Figure 4:**
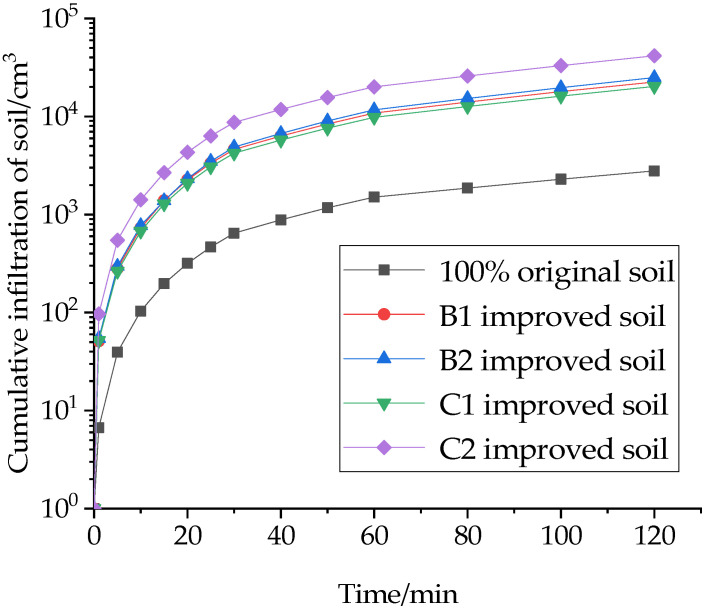
Effect of ameliorants on cumulative soil infiltration.

**Figure 5:**
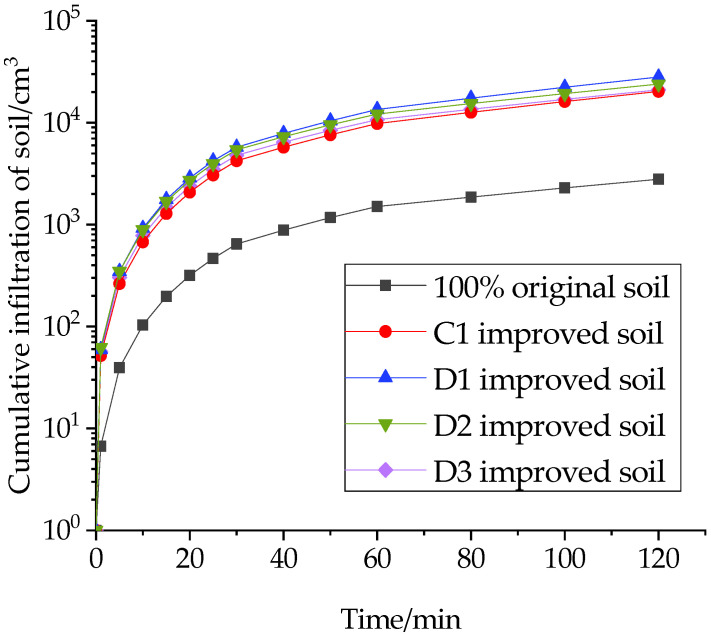
Effect of structural ameliorants on cumulative soil infiltration.

**Figure 6:**
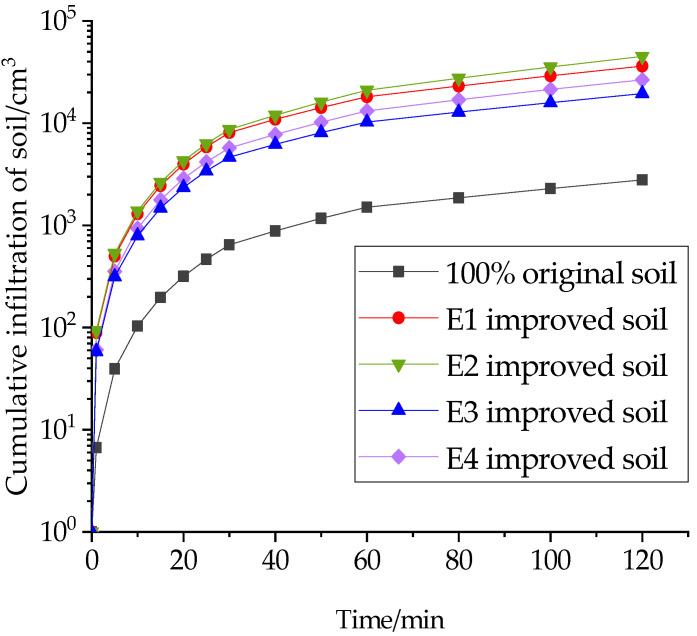
Effect of the interweaving of ameliorants and structural amendments on cumulative soil infiltration.

Under the related action of gravitational potential and substrate potential, water from rainfall and irrigation seeps from the soil surface into the soil interior and becomes soil water, which is a dynamic process of the mutual transfer and loss of surface water and groundwater. In this study, the cumulative soil infiltration for the first 120 min was chosen to measure the infiltration capacity of the improved soil, as shown in Figure 4, Figure 5 and Figure 6.

As shown in Figure 4, the cumulative soil infiltration rate tends to increase with the increase in the specific gravity of the ameliorant. The cumulative infiltration rates of 10% sand and 20% sand alone were 8 and 8.9 times higher than those of the original soil, and the cumulative infiltration rates of 20% and 30% grain shells alone were 7.3 and 14.9 times higher than those of the original soil. This shows that the increase in the ameliorant can improve the internal infiltration capacity of the soil. It makes more pore space inside the soil, thus making the modified soil have better infiltration ability. Meanwhile, the amendment effect of grain shells was significantly improved compared to sand with the same percentage increase, which is because the volume of grain shells is larger than that of sand and has a larger proportion in the amended soil, so the change in its content has a greater effect on the infiltration amount.

The addition of structural ameliorants improved the cumulative infiltration of the soil to different degrees. As shown in Figure 5, the cumulative infiltration volume of soil increased by 38.3% when FGD gypsum was added together with 20% grain shells, while the enhancement effect of adding PAM was not obvious, and the cumulative infiltration volume even showed a certain decrease when PAM was added together with FGD gypsum. This is because PAM has strong water absorption, and under the long-term soaking of water, the internal structure of the soil changed from granular to multibranched fibrous, which inhibited the infiltration capacity of the modified soil.

On this basis, sand was added to study the effect of the inorganic ameliorant and structural ameliorant on the cumulative infiltration of soil. Figure 5 and Figure 6 demonstrate that the addition of sand has a substantial impact on the cumulative infiltration rate. Specifically, incorporating 10% sand and 20% grain shells results in a 78.3% increase in cumulative infiltration compared to grain shells alone. Moreover, the addition of PAM leads to an 18.4% decrease in the cumulative infiltration of the modified soil mixed with sand and grain shells, confirming that PAM is less effective than other soil ameliorants in enhancing infiltration capacity. Additionally, incorporating both PAM and FGD gypsum leads to a 26.7% increase in cumulative infiltration. These findings highlight the significant influence of sand on the infiltration capacity of amended soil.

**Figure 7:**
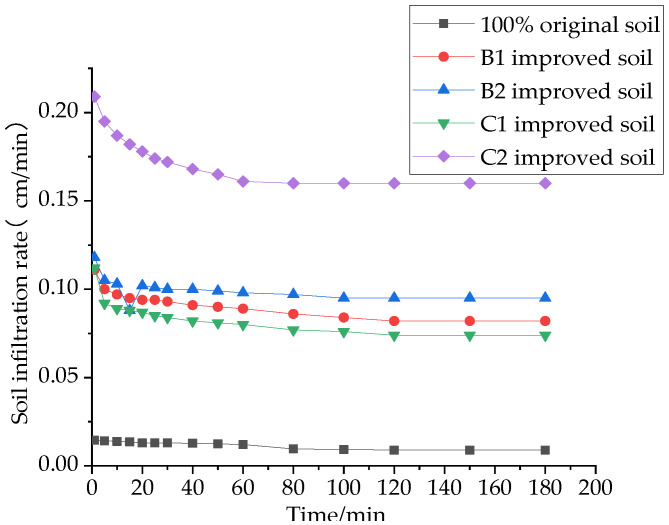
Effect of ameliorants on the soil infiltration rate.

**Figure 8:**
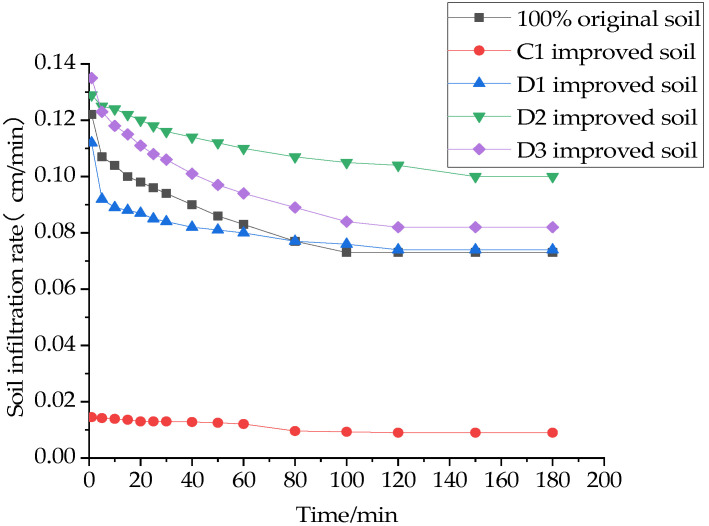
Effect of structural ameliorants on the soil infiltration rate.

**Figure 9:**
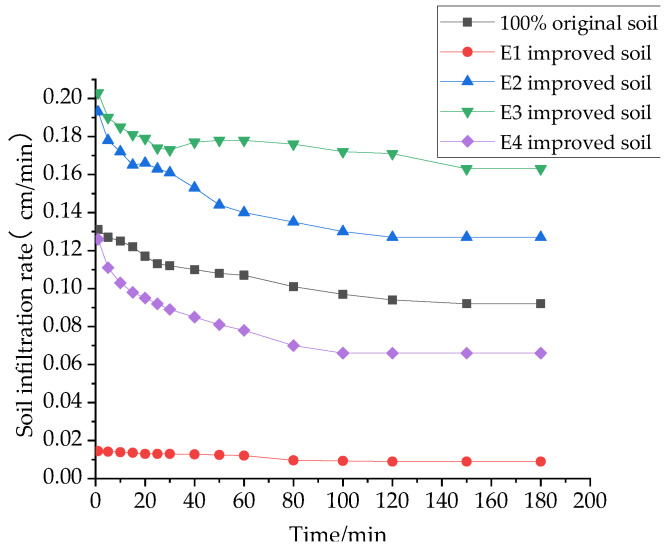
Effect of the interweaving of amendments and structural ameliorants on the soil infiltration rate.

The infiltration rate of water in the soil is a quantitative representation of infiltration capacity and an important indicator for evaluating the infiltration capacity of the soil. The effects of different materials on the soil infiltration rate are shown in Figure 7, Figure 8 and Figure 9.

It was found that the soil infiltration rate showed a decreasing trend. It was first higher in the initial stage, and it decreased faster in the first 60 min. In the second 60 min, it entered into a slowly decreasing state. It reached a stable infiltration state at about 120 min. This is because, at the beginning, there are a large number of pores inside the soil, and with the infiltration of water, most of the pores are filled to fullness, and the space where the soil can hold water is reduced, resulting in the infiltration capacity of the soil becoming weaker and the infiltration rate of the soil becoming smaller. Therefore, the stable infiltration rate of 180 min, which is more representative, is selected as the evaluation index in this paper.

It can be seen in Figure 7 that the stable infiltration rate of the soil mixed with 10% sand and 20% sand increased by 9.1 and 10.6 times compared to the original soil, and the stable infiltration rate of the soil mixed with 20% and 30% grain shells increased by 8.2 and 17.8 times compared to the original soil. This is consistent with the conclusion that the increase in the specific gravity of the ameliorant increased the cumulative infiltration of the amended soil, as mentioned above.

As can be seen in Figure 8 and Figure 9, the effects of the single addition of a structural ameliorant or interwoven structural ameliorant and ameliorant on the stable infiltration rate of the soil are consistent with the previous subsection; in both cases, the addition of FGD makes the stable infiltration rate increase by 135% and 128%, respectively, while the addition of PAM is less effective.

In this study, we utilized the Kostyakov infiltration model to evaluate the infiltration capacity of the modified soil. In the Kostiakov infiltration model, the *p* value reflects the initial infiltration capacity of the modified soil. The greater the *p* value, the stronger the initial infiltration capacity of the modified soil. The *β* value reflects the infiltration attenuation rate of the improved soil. The greater the *β* value, the slower the infiltration attenuation rate. As shown in Table 5, in the case of a single sand and grain shells, with the increase in the proportion of sand and grain shells, the *p* value and *β* value also increase, indicating that the initial infiltration capacity and infiltration attenuation rate of the improved soil have been improved.

By adding PAM ameliorant to the soil, the *p* value and *β* value decreased slightly, indicating that the addition of PAM did not help to improve the infiltration capacity of the soil. The *p* value and *β* value of the improved soil were significantly improved by adding desulfurized gypsum under the condition of single-doped grain shells or PAM, indicating that the infiltration capacity of the improved soil with desulfurized gypsum was modified, and the *β* value of desulfurized gypsum was added. Compared with adding PAM or adding two structural ameliorants at the same time, it is the largest, indicating that this situation can slow down the attenuation rate of soil infiltration, thereby increasing the cumulative infiltration of soil, which also verifies that adding desulfurized gypsum has a good effect.

### 3.2. Soil Pore Characteristics Analysis

**Figure 10:**
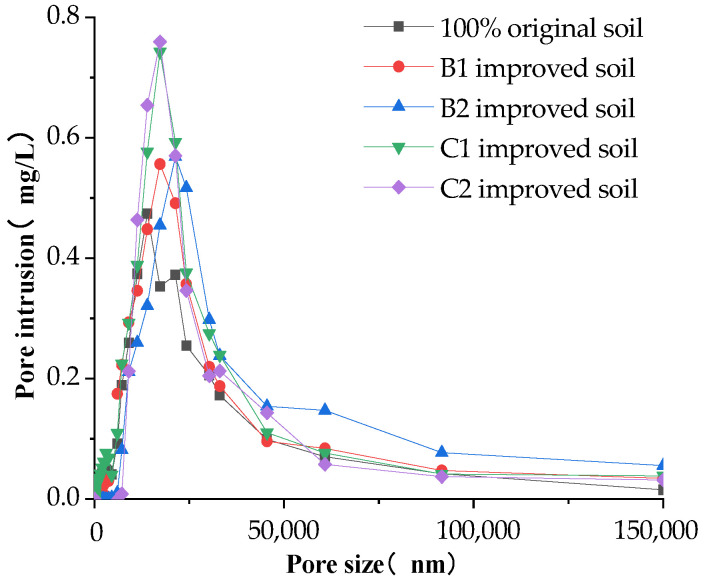
Pore distribution of soil amended with ameliorants.

**Figure 11:**
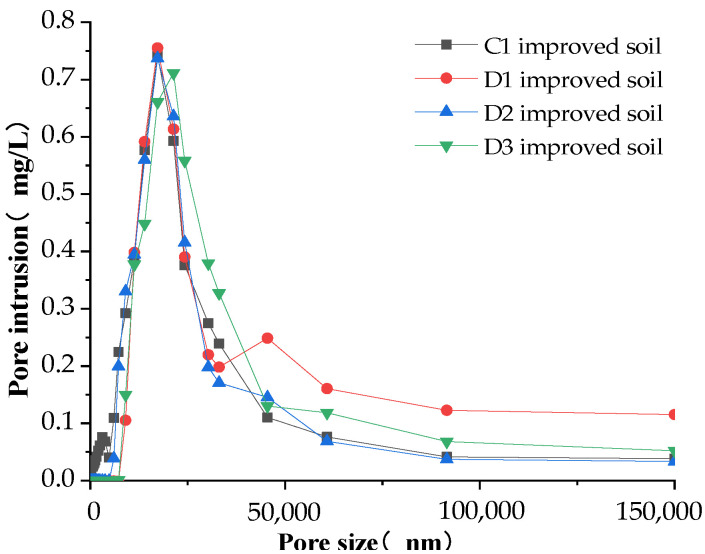
Pore distribution of soil amended with structural ameliorant.

**Figure 12:**
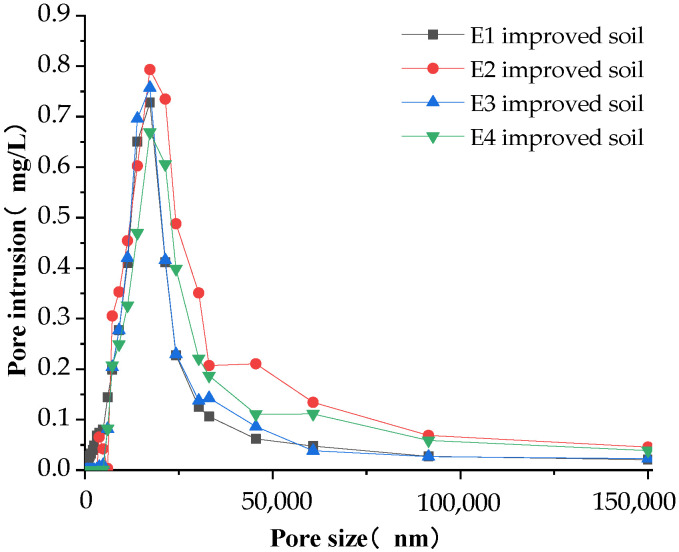
The pore distribution of soil amended by the interweaving of ameliorants and structural ameliorants.

Soil is divided into three categories according to the size of the soil pore size. The pore size that is greater than 100 μm is defined as the large pore, also known as the “aeration pore” or “non-capillary pore”. This kind of soil has good aeration and drainage [29,30,31]. When the pore size is between 20 and 100 μm, it has good hydraulic conductivity and fast capillary water movement. Meanwhile, when the pore size is between 3 and 20 μm, it is defined as small pores, which usually have good water-holding capacity but slow capillary water movement. The pore distribution characteristics of the modified soil measured by the mercury-pressure method are shown in Figure 10, Figure 11 and Figure 12.

In general, the amount of pore invasion content (PIC) was rising and then decreasing when the pore diameter kept increasing, with a peak at the pore diameter of 25,000 nm (25 μm). The amount of invasion leveled off when the pore diameter was greater than 100,000 nm (100 μm), indicating that most of the pores in the modified soil are mainly distributed by the medium pores of 25 μm, while there is less distribution of large pores greater than 100 μm.

In Figure 10, it can be seen that the maximum pore intrusion is 0.75 mg/L for grain shells alone and 0.55 mg/L for sand alone. Under the same conditions, the water infiltration capacity of the modified soil mixed with grain shells is better than that of sand. The maximum pore intrusion of 20% sand alone increased by 3% compared to 10% sand alone, and the maximum pore intrusion of 30% grain shells alone increased by 2% compared to 20% grain shells alone. Both of them were greater than the maximum pore intrusion of the original soil. This verifies the conclusion in Section 3.1 that the stable infiltration rate of either sand or shells alone is greater than that of the original soil sample, and the stable infiltration rate of the soil is improved with the increase in the percentage of improved materials.

As shown in Figure 11, the overall trend of the pore distribution characteristics of the soil after the addition of the structural ameliorant was similar, but in the modified soil, after adding FGD gypsum, the second and third peaks appear at 50 μm and 350 μm, indicating that the modified soil with FGD gypsum had more medium pores of 50 μm and larger pores of 350 μm distributed than the other three modified soils. The maximum pore intrusion of the amended soil with the simultaneous addition of FGD gypsum and PAM was the lowest: about 0.7 mg/L. This indicated that the simultaneous addition of two structural ameliorants had no significant effect on the improvement of the soil. After the second peak at 50 μm, the pore intrusion of the amended soil with FGD gypsum was almost higher than the other three amended soils, indicating that the amended soil with FGD gypsum had the best improvement effect, which also verified the conclusion obtained in Section 3.1: the stable infiltration rate of the soil with FGD gypsum increased by 135% under the condition that the soil was mixed with 20% grain shells alone.

As shown in Figure 12, under the influence of the interweaving of ameliorants and structural ameliorants, soil pore distribution characteristics showed an increasing trend followed by a decreasing trend. In the case of mixing 10% sand and 20% grain shells, the maximum pore intrusion was 0.8 mg/L with FGD gypsum, 0.78 mg/L with PAM, 0.68 mg/L with both FGD gypsum and PAM and 0.75 mg/L with no structural ameliorant. After the second peak at 50 μm, the pore intrusion was almost higher than that of the other three modified soils, which indicated that FGD gypsum was a better structural ameliorant and could improve the infiltration performance.

### 3.3. Pollutant Removal Capability

**Figure 13:**
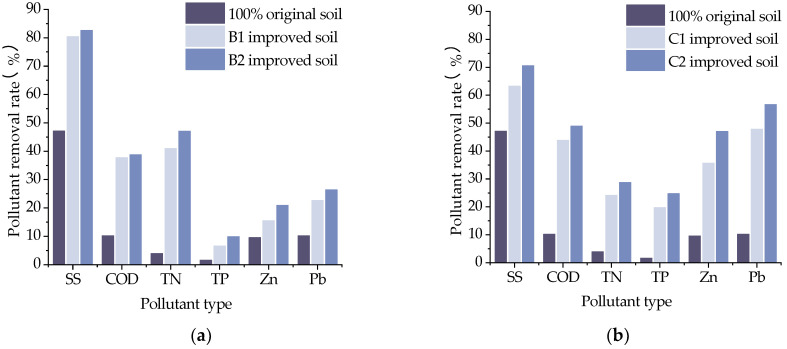
Pollution removal rate of soil amended with ameliorants: (**a**) Sand-amended soil; (**b**) Grain shells-amended soil.

**Figure 14:**
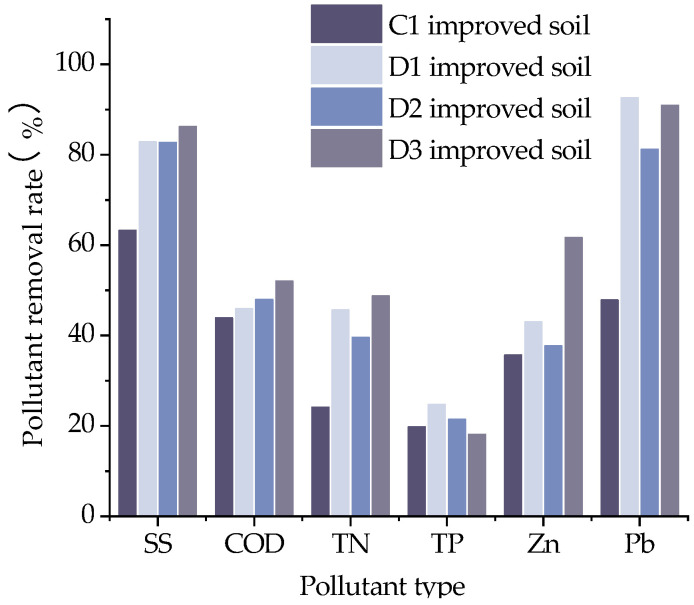
Pollution removal rate of soil amended with structural ameliorants.

**Figure 15:**
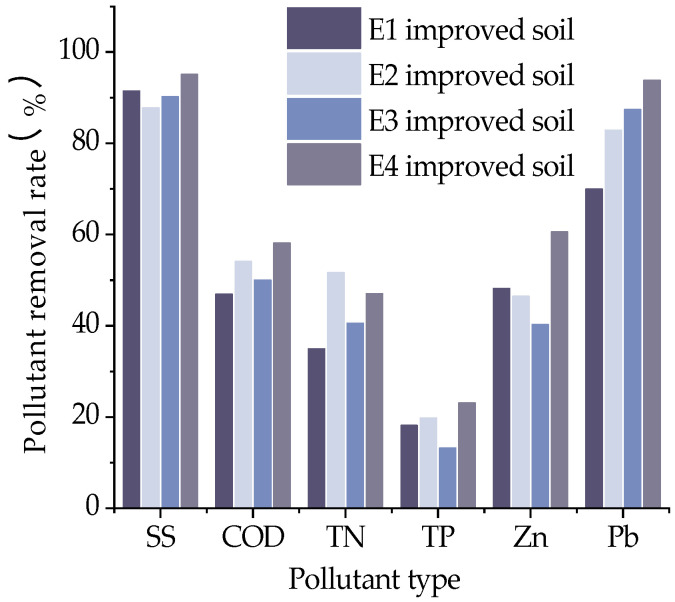
Pollution removal rate of soil amended with the interweaving of ameliorants and structural ameliorants.

Soil pollutants mainly include organic and inorganic pollutants. When the soil contains too many pollutants that exceed its self-purification capacity, it will cause changes in the soil composition, structure and function, and the pollutants in the soil and the harmful products generated from their decomposition can directly or indirectly affect human health [32,33,34,35]. Different amendment materials were selected to improve the contaminated soil and analyze its purification effect on pollutants. The pollutant removal rates of different amended soils are shown in Figure 13, Figure 14 and Figure 15.

The decontamination ability of the original soil is relatively weak, and the addition of the amendment material can effectively improve the pollutant removal rate of the soil. In Figure 13, it can be seen that sand can effectively improve SS-type pollution and TN pollution, and the removal rate of pollutants increased by 70.6% compared with the original soil and increased by nearly 15% compared with that of grain shells alone. When mixed with sand of different proportions, the removal rate does not change significantly, because SS pollution mainly contains insoluble sediment with a particle size of 450 nm, and sand can filter out the insoluble particles in the pollutants. It can be seen in the pore characteristic distribution diagram that the PIC of sand at the 450 nm pore is higher than that of other improved materials, so the sand has a good removal effect on SS and TN pollution. The incorporation of grain shells significantly improved the removal rate of Pb and Zn by 367% and 272%, respectively, compared with the original soil and by 81.1% and 70.6%, respectively, compared with sand alone. It also improved the COD and TP pollution, and the pollutant removal rate appeared to be significantly increased with the increase in admixture. This is because the grain shells, with their good adsorption and cellulose, can filter out most of the pollutants, so the removal of the heavy metals Zn, Pb, COD and TP by the grain shells is better.

Overall, SS pollution and heavy metal Pb pollution are easier to remove. As shown in Figure 14 and Figure 15, the removal rates of SS by adding FGD gypsum alone and PAM alone to the grain shells were the same, with an improvement of about 31% over that without the structural ameliorant, while the removal rate of SS by adding both FGD gypsum and PAM to the grain shells increased by about 36.4% compared to that without the structural ameliorant. The increasing extent of the COD removal rate by adding the structural ameliorant was not so obvious, and the average increase was only about 12.4%. The addition of FGD gypsum, a structural ameliorant, improved the purification effect of both TN and TP, increasing the removal rate of TN by about 44.64% and the removal rate of TP by about 25%. For metallic stormwater runoff, the removal rates of Zn and Pb by adding FGD gypsum increased by about 20.5% and 93.5%, respectively, compared with no structural ameliorant; the removal rates of Zn and Pb by adding PAM increased by about 5.6% and 69.6%, respectively, compared with no structural ameliorant; the removal rates of Zn and Pb by adding both FGD gypsum and PAM increased by about 72.8% and 90%, respectively, compared with no structural ameliorant. The removal of Zn by adding FGD gypsum was better, and the removal of Pb by adding FGD gypsum and PAM at the same time was better.

Adding FGD gypsum together with 10% sand and 20% grain shells increased the removal rate of COD by 15.2%, adding PAM increased the removal rate of COD by about 6.5% and adding both FGD gypsum and PAM increased the removal rate of COD by about 24.0%, so for COD-type stormwater runoff, adding both FGD gypsum and PAM to the modified soil can achieve better removal results. For heavy metal-type stormwater runoff, the modified soil with the simultaneous addition of FGD gypsum and PAM increased the removal rates of Zn and Pb by about 25.7% and 34.0%, respectively. Therefore, for interwoven soils, the simultaneous addition of FGD gypsum and PAM is required to improve the purification capacity, and the infiltration effect of modified soils with the addition of FGD gypsum is obvious.

## 4. Conclusions

This study investigated the infiltration and decontamination capacities of different modified soils using inorganic amendments (sand and grain shells) and structural amendments (PAM and FGD gypsum). The infiltration tests, pore distribution determination and decontamination tests were used to accomplish this goal. The following conclusions can be drawn:

(1) The addition of ameliorants significantly improved the infiltration capacity of the soil, with grain shells showing a better improvement effect compared to sand under the same conditions. The addition of FGD gypsum effectively increased the soil infiltration capacity and slowed down the rate of infiltration attenuation. PAM was not as effective as other modification materials in enhancing infiltration capacity. The optimal infiltration capacity was achieved when inorganic modifiers and FGD gypsum were mixed.
(2) The MIP test results show that pores in the soil were mainly composed of 25 μm medium-sized pores. With the addition of the amendments, the soil porosity was significantly increased. The modified soil with grain shells alone had a higher porosity compared to soil with sand. The addition of FGD gypsum to the modified soil resulted in more 50 μm medium-sized pores and 350 μm large pores compared to the other three modified soils, indicating that it led to the most effective improvement of the infiltration capacity.
(3) Based on an analysis of typical pollutants in rainwater in Yangzhou, the soil decontamination test was conducted to assess the decontamination capacity of various modified soils. Grain shells exhibited excellent adsorption properties due to their high cellulose content, effectively removing a wide range of pollutants. Sand demonstrated a good removal efficacy for suspended SS and total TN, reaching saturation at a mixing ratio of 10%. FGD presented good pollutant reduction for TN and TP, while the combination of PAM and FGD gypsum exhibited excellent performance for COD.
(4) In highly polluted areas, a proportioning scheme consisting of 20% grain shells, 10% sand, 0.5 g/kg FGD gypsum and 0.1 g/kg PAM (referred to as the E4 proportioning scheme) is recommended due to its superior infiltration and decontamination capacities. For areas with high permeability requirements, a proportioning scheme consisting of 20% grain shells, 0.5 g/kg FGD gypsum and 0.1 g/kg PAM (referred to as the D3 proportioning scheme) is suggested.

According to the study findings, the use of soil amendments enhances soil infiltration and decontamination capacities, enabling soil improvement and environmental protection. Compared to traditional soil amendment methods, incorporating soil amendments exhibits greater adaptability and stability. This can provide novel pathways and options for various domains, including urban green space development, soil remediation and soil and water conservation, and holds tremendous potential for widespread application.

In the study, only two types of inorganic amendments (i.e., sand and grain shell) and structural ameliorants (i.e., desulfurization gypsum and polyacrylamide) were considered. In future research, it would be worthwhile to consider additional variables (e.g., other types of ameliorants, contents and land use types).

## Figures and Tables

**Table 1:** Properties of the soil.

	Soil Component			Physical Properties				
	Sand	Silt	Clay	Density (g/cm^3^)	Capacity (N/cm^3^)	Moisture Content (%)	Void Ratio (%)	pH Value
Particle size range (mm)	2~0.02	0.02~0.002	<0.002	1.10	10.78	14.34	38.52	6.70
Percentage (%)	81.49	14.92	3.59					

**Table 2:** Physical properties of different modified materials.

Ameliorants	Density (g/cm^3^)	Unit Weight (N/cm^3^)
Sand	1.513	14.8374
Grain shells	0.118	1.1564
Polyacrylamide (PAM)	1.289	12.6322
FGD gypsum	1.665	16.317

**Table 3:** Different proportioning schemes of modified materials.

Number	Different Ratio Combinations of Improved Materials
A0	100% original soil
B1	90% soil + 10% sand
B2	80% soil + 20% sand
C1	80% soil + 20% grain shells
C2	70% soil + 30% grain shells
D1	80% soil + 20% grain shells + 0.5 g/kg FGD gypsum
D2	80% soil + 20% grain shells + 0.1 g/kg PAM
D3	80% soil + 20% grain shells + 0.5 g/kg FGD gypsum + 0.1 g/kg PAM
E1	70% soil + 10% sand + 20% grain shells
E2	70% soil+ 10% sand + 20% grain shells + 0.5 g/kg FGD gypsum
E3	70% soil + 10% sand + 20% grain shells + 0.1 g/kg PAM
E4	70% soil + 10% sand + 20% grain shells + 0.5 g/kg FGD gypsum + 0.1 g/kg PAM

**Table 4:** Detection of rainwater runoff pollutants and the corresponding reagent addition.

Pollutant Type	Determination Method	Concentration	Reagents Used	Mass Required for 100 L (g)
SS	Gravimetric method	420	Road deposit soil	60.019
COD	Potassium dichromate method	400	C_6_H_12_O_6_	42.956
TN	Potassium persulfate oxidation UV spectrophotometry	8.0	NH_4_Cl	4.828
TP	Ammonium molybdate spectrophotometric method	0.5	KH_2_PO_4_	0.184
Zn	Atomic absorption spectrophotometry for heavy metals	3.0	Zn(NO_3_)	0.238
Pb		0.5	Pb(NO_3_)	0.061

**Table 5:** Parameters of the Kostiakov infiltration model for different proportions of modified soil.

Different Soil Groups		lgI=P+β×lgt		
		*p*	*β*	*R* ^2^
Raw soil	A0	0.77	1.32	0.9932
Single-doped sand or grain shells	B1	1.62	1.33	0.9938
	B2	1.64	1.339	0.9935
	C1	1.62	1.308	0.9931
	C2	1.91	1.32	0.9945
Single-doped sand and structural ameliorants	D1	1.71	1.339	0.9946
	D2	1.73	1.298	0.9939
	D3	1.69	1.293	0.9933
Mixed with sand, grain shells and structural ameliorants	E1	1.89	1.31	0.9937
	E2	1.89	1.345	0.9946
	E3	1.71	1.269	0.9936
	E4	1.72	1.324	0.9946

*R*^2^ is the coefficient of determination of the linear regression model.

## Data Availability

Not applicable.
